# *FBN1* TB5 domain variants in acromelic dysplasia: multisystem manifestations, genotype–phenotype correlations, and partial responses to growth hormone therapy

**DOI:** 10.3389/fendo.2026.1883383

**Published:** 2026-07-22

**Authors:** Jun Zhang, Meng-Tian Huang, Bing Wang, Yan-Yan Lin, Ru-Jiang Zheng, Huang-Meng Xiao, Hua-Mei Ma, Song Guo, Qiu-Li Chen, Yan-Hong Li

**Affiliations:** 1 Department of Pediatrics, The First Affiliated Hospital, Sun Yat-sen University, Guangzhou, China; 2 Department of Pediatrics, Peking University Shenzhen Hospital, Shenzhen, China; 3 Department of Child Health, Department of Pediatrics, Women and Children’s Hospital, School of Medicine, Xiamen University, Xiamen, China

**Keywords:** acromicric dysplasia, *FBN1* gene, geleophysic dysplasia 2, genotype–phenotype correlation, growth-promoting therapy, TB5 domain

## Abstract

**Background:**

Acromelic dysplasias, including acromicric dysplasia (AD) and geleophysic dysplasia type 2 (GD2), are ultrarare disorders caused by *FBN1* variants in the transforming growth factor-β-binding protein-like domain 5 (TB5). These conditions are characterized by severe short stature and variable multisystem involvement, but genotype–phenotype correlations and treatment responses remain incompletely defined.

**Objective:**

To characterize the clinical spectrum, genotype–phenotype correlations, and growth outcomes in a Chinese cohort of patients with *FBN1*-related AD/GD2.

**Methods:**

We conducted a retrospective analysis of 14 patients with AD or GD2 from eight unrelated families. Clinical, radiographic, and genetic data were collected. Genotype–phenotype correlations were further explored by integrating data from two previously published cohorts (n = 52). Growth outcomes were evaluated in patients receiving recombinant human growth hormone (rhGH) therapy.

**Results:**

All patients exhibited severe short stature (mean height-SDS of -5.28 ± 1.13) with brachydactyly and delayed bone age. Multisystem involvement, including skeletal abnormalities, airway and otologic complications, skin thickening, joint limitation, hepatomegaly, and cardiopulmonary disease (50.0%, exclusively on GD2), was common. All the variants were heterozygous missense mutations clustered in the TB5 domain, including two novel *de novo* variants. Genotype–phenotype preliminary analysis revealed a greater mortality in patients with GD2 than in patients with AD (29.6%(15.9 -48.5%) vs. 4.0%(0.7-19.5%), P = 0.038). Variants with cysteine or aromatic residues substitutions were associated with increased mortality compared with that associated with other substitutions (overall *P* < 0.001). Eleven patients received growth-promoting therapy. Among the eight patients treated at our center, growth-promoting therapy maintained a growth velocity during the first two years (approximately 6 cm/year) but declined thereafter, with no significant improvement in height-SDS over a mean treatment duration of 3.6 years.

**Conclusion:**

*FBN1*-related AD/GD2 represent multisystem disorders rather than isolated skeletal dysplasias. Variants affecting cysteine or aromatic residues in the TB5 domain maybe associated with worse outcomes. Growth-promoting therapy may contribute to maintaince of growth velocity but catch-up growth was not clearly observed. These findings suggest that molecular stratification may be helpful for risk assessment and clinical decision-making, pending further validation.

## Introduction

Since the identification of *FBN1* (HGNC: 3603) as the causative gene for Marfan syndrome in 1991 (1), pathogenic variants in *FBN1* have also been implicated in a distinct group of autosomal dominant acromelic dysplasias, including acromicric dysplasia (AD; OMIM #102370) and geleophysic dysplasia type 2 (GD2; OMIM #614185), which represent contrasting phenotypes to those of Marfan syndrome (2). AD is an ultrarare skeletal dysplasia characterized by severe disproportionate short stature, brachydactyly, characteristic craniofacial features, and a pseudomuscular habitus and is often accompanied by joint contractures and carpal tunnel syndrome (2). GD2 is a progressive multisystem disorder with overlapping skeletal features and additional cardiopulmonary involvement, including valvular thickening and tracheal stenosis (3, 4).

Pathogenic variants associated with AD and GD2 are consistently clustered within the transforming growth factor-β-binding protein-like domain 5 (TB5) of fibrillin-1 (2). In growth plate cartilage, fibrillin-1 plays a key role in regulating extracellular matrix integrity and modulating TGF-β signaling, which is essential for chondrogenesis and longitudinal bone growth (5).

However, the current knowledge remains limited by small number of case series and isolated reports, and there is a paucity of systematic data concerning longitudinal phenotypes, genotype–phenotype correlations, and the efficacy of growth-promoting interventions in affected patients. In particular, the response to growth-promoting therapy in this population has not been well defined. To address these gaps, we retrospectively analyzed 14 patients with *FBN1*-related AD/GD2 from a single center, including a subgroup who received growth-promoting therapy, to better delineate clinical heterogeneity and treatment outcomes.

## Materials and methods

### Study subjects

This retrospective single-center study was conducted at the Growth and Development Center, Department of Pediatrics, The First Affiliated Hospital of Sun Yat-sen University. Fourteen participants who were diagnosed with AD or GD2 due to *FBN1* variants between January 2011 and January 2021 were included. Clinical data were obtained from electronic and paper medical records. The study was performed in accordance with the principles of the Declaration of Helsinki (2013 revision) and approved by the Ethics Committee of The First Affiliated Hospital, Sun Yat-sen University [approval no (2025).541].

### Z score calculation

Height and weight Z scores were calculated according to the 2009 growth reference standards for Chinese children and adolescents (6), whereas body mass index (BMI) Z scores were derived from the corresponding age- and sex-specific BMI reference standards published in the same year (6).

### Genetic analysis

Written informed consent was obtained from all participants or their legal guardians. Peripheral blood (2 mL) collected in EDTA anticoagulant tubes was used for genetic analysis. Whole-exome sequencing was performed by KingMed Diagnostics Group Co., Ltd. (Guangzhou, China), and candidate variants were confirmed by PCR–Sanger sequencing. Variant interpretation followed American College of Medical Genetics and Genomics (ACMG) guidelines, and inheritance was assessed by familial cosegregation analysis.

### Genotype–phenotype correlation analysis

To explore genotype–phenotype associations, we analyzed two previously published longitudinal cohorts of patients with *FBN1*-related AD/GD2 (Le Goff et al. (2); Marzin et al. (7)). To minimize potential bias from intrafamilial clustering, only one affected individual (the proband) per family was included when multiple family members were reported. To ensure consistency across datasets, we performed a systematic re-extraction and harmonization of individual-level data from all included published cohorts. All original manuscripts and Supplementary Materials were independently reviewed, and detailed clinical and genetic information was manually curated for each patient, including exact *FBN1* variant description, corresponding amino acid change, clinical phenotype, and mortality outcome. Variant nomenclature was standardized according to Human Genome Variation Society (HGVS) recommendations based on the original reported coding sequences and protein annotations. When discrepancies in reporting existed across studies, the original variant descriptions were cross-checked against the published context and unified under a consistent annotation framework. For clinical variables, we extracted only explicitly reported data, and missing variables were handled by pairwise deletion in analyses. The denominator for each analysis was therefore variable-dependent and was clearly specified in the corresponding tables and figures. This approach allowed maximization of data utilization while maintaining transparency regarding incomplete reporting across studies.

TB5 variants were categorized according to their biochemical impact, including (1) cysteine-altering variants (loss or gain of cysteine residues), (2) aromatic amino acid substitutions (e.g., tyrosine and phenylalanine), and (3) other substitutions.

### Growth-promoting therapy

Eight participants received recombinant human growth hormone (rhGH) at 50–66.7 μg/kg/day. Four participants also received a gonadotropin-releasing hormone analog (GnRHa; triptorelin, 80–150 μg/kg every 4 weeks) because of advanced bone age, and two received stanozolol (20–40 μg/kg/day). Compliance was defined as poor if patients missed more than three doses per week on average during treatment, based on caregiver/patient reports and medical records.

### Statistical analysis

The normality of the continuous variables was assessed using the Shapiro–Wilk test. Normally distributed data are presented as the mean ± SD and were compared using paired or unpaired Student’s t tests; nonnormally distributed data are presented as the median (IQR) and were analyzed using the Wilcoxon signed-rank or Mann–Whitney U test. Mortality rates were compared across groups. Subgroup comparisons were performed using Fisher’s exact test, with Bonferroni correction applied for multiple pairwise comparisons. The 95% confidence intervals for mortality rates were calculated using the Wilson method. Statistical analyses were performed with SPSS v26.0 (IBM, Chicago, IL, USA). Survival analyses and Kaplan–Meier curves were generated using Prism 9.0. A two-sided P value < 0.05 was considered statistically significant.

## Results

### Clinical characteristics

**Figure 1:**
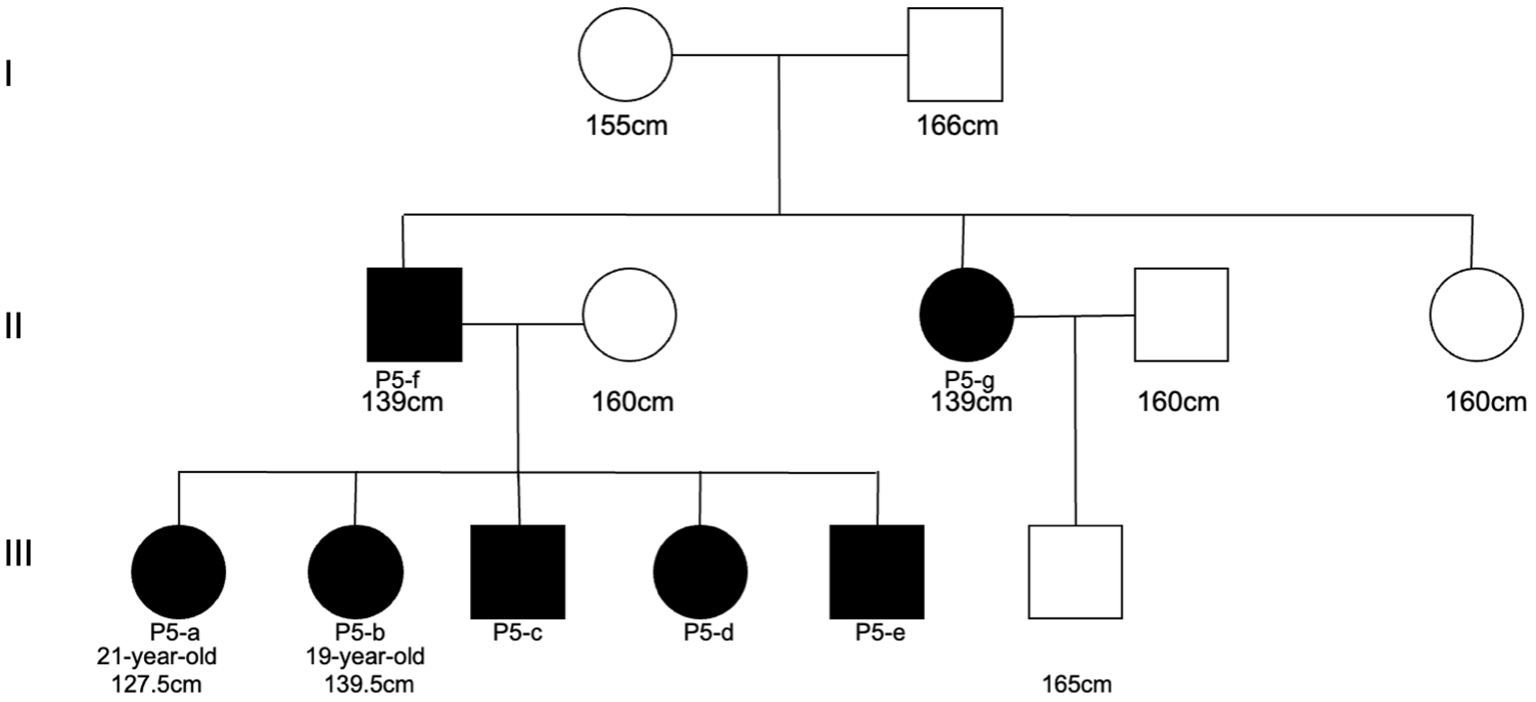
Pedigree of family P5.

This study included 14 participants with AD or GD2 from eight unrelated Han Chinese families (8 males and 6 females). Individuals P5-a to P5-g belonged to a single extended pedigree (**Figure 1**), whereas the remaining participants were from distinct families (**Table 1**). The median gestational age was 37.3 weeks (IQR, 37–38), with a mean birth length of 49.7 ± 1.6 cm and a median birth weight of 2.9 kg (IQR, 2.6–3.0). Most participants (11/13, 84.6%) exhibited growth retardation before age 3 years.

**Table 1:** Clinical characteristics of the 14 AD/GD2 participants.

Participant	Sex	Birth			Initial presentation at our center						OAGD	Limited joint mobility	Cardiac/Pulmonary involvement	FBN1 gene variation		DX
		GA (weeks)	Ht (cm)	Wt (kg)	Age (yrs)	Ht(cm)/SD	HC (cm)	Wt (kg)	BMI (kg/m2)/SD	BA (yrs)				cDNA	Protein	
P1	M	38	48	2.8	4.56	80.0/-6.90	51.0	10.0	15.63/0.35	NA	NP	Interphalangeal, elbow, and knee joints.	Severe mitral valve stenosis and severe pulmonary hypertension; underwent surgery at 3 years of age.	c.5096A>G	Tyr1699Cys	GD2
P2	M	38	48	3.2	4.46	82.8/-6.07	49.0	11.0	16.04/0.70	2.5	1 year	Left metacarpophalangeal and interphalangeal joints.	No cardiopulmonary involvement was observed.	c.5284G>A	Gly1762Ser	AD
P3	M	39	51	3.5	8.68	109.5/-4.23	NA	29.5	24.60/4.19	5.5	3 years	Stenosing tenosynovitis of the right middle and ring fingers was diagnosed at age 5 and surgically released at age 8.	Severe pulmonary hypertension, currently managed with medication.	c.5284G>A	Gly1762Ser	GD2
P4	M	41	50	3	10.24	108.1/-5.29	50.5	20.0	17.12/-0.01	9.5	NP	Metacarpophalangeal joints and interphalangeal joints.	No cardiopulmonary involvement was observed.	c.5250T>G	Ser1750Arg	AD
P5-a	F	37	46	2.5	14.07	119.9/-6.81	52.0	25.0	17.39/-0.62	13.25	NP	No joint involvement.	No cardiopulmonary involvement was observed.	c.5099A>G	Tyr1700Cys	AD
P5-b	F	38	50	2.9	12.38	120.7/-5.32	52.0	23.5	16.13/-0.71	12.75	NP	No joint involvement.	No cardiopulmonary involvement was observed.	c.5099A>G	Tyr1700Cys	AD
P5-c	M	40	50	3	7.20	100.4/-4.82	49.0	18.0	17.86/1.30	5.5	NP	No joint involvement.	Malformations of the aorta and ascending aorta.	c.5099A>G	Tyr1700Cys	GD2
P5-d	F	40	50	3	4.70	88.6/-4.85	48.0	13.0	15.87/1.20	4	NP	No joint involvement.	Malformations of the aorta and ascending aorta.	c.5099A>G	Tyr1700Cys	GD2
P5-e	M	38	51	3	3.93	86.5/-4.36	49.0	11.5	15.37/0.03	3	NP	No joint involvement.	No cardiopulmonary involvement was observed.	c.5099A>G	Tyr1700Cys	AD
P5-f	M	NA	NA	NA	43.00	139.0/-5.52	54.5	48.0	24.84/1.33	NA	NA	No joint involvement.	Pulmonary valve stenosis was diagnosed at age 40 years, and balloon valvuloplasty was performed.	c.5099A>G	Tyr1700Cys	GD2
P5-g	F	NA	NA	NA	41.00	139.0/-4.0	NA	NA	NA	NA	NA	No joint involvement.	No cardiopulmonary involvement was observed.	c.5099A>G	Tyr1700Cys	AD
P6	F	40	51	3.9	8.16	89.3/-7.43	51.2	13.18	16.53/0.74	3	1 year	Metacarpophalangeal, interphalangeal, knee, and elbow joints.	The participant had mixed ventilatory dysfunction with recurrent pulmonary infections.	c.5159G>C	Cys1720Ser	GD2
P7	M	40	50	3.9	11.35	120.0/-4.06	53.0	30.5	21.18/1.35	9.5	6 years	Metacarpophalangeal joints and interphalangeal joints.	No cardiopulmonary involvement was observed.	c.5285G>A	Gly1762Asp	AD
P8	F	40	51	3	6.24	98.3/-4.28	52.0	16.5	17.08/1.49	6	3 years	No joint involvement.	Pulmonary valve stenosis was diagnosed and treated with balloon dilation at age 6, resulting in good recovery.	c.5099A>G	Try1700Cys	GD2

NA, not available. M, Male. F, female. GA, gestational age. Ht, height. Wt, weight. HC, Head circumference. BMI, body mass index. BA, bone age. OAGD:onset age of growth deceleration. NP, Neonatal period. DX, diagnosis.

At the initial evaluation at our center, participants with AD or GD2 had a median chronological age of 8.4 years (IQR, 4.7–12.8) and a mean bone age (BA) of 6.77 ± 3.88 years. The mean height-SDS was -5.28 ± 1.13. Overall, 64.3% (9/14) were Tanner stage I. The median difference in height–arm span was 5.0 cm (IQR, 3.25–8.75), and the arm span-to-height ratio was 0.96 (IQR, 0.92–0.98). The mean sitting height-to-height ratio was 0.60 ± 0.02. The mean BMI was 18.13 ± 3.28 kg/m², and the median BMI-SDS was 0.74 (IQR, 0.01–1.34), with 46.2% (6/13) of the participants classified as overweight or obese. BMI-SDS differed between groups, with median values of 0.01 (IQR, −0.64 to 0.86) in patients with AD and 1.30 (IQR, 0.74–1.49) in patients with GD2 (*P* = 0.03). The mean occipitofrontal circumference was 50.9 ± 1.9 cm, and cognitive function was normal in all participants.

In Family P5, there were seven affected individuals sharing the identical genotype, all carrying the *FBN1* c.5099A>G (p.Tyr1700Cys) variant. Among them, subject P5-c, at 7.2 years of age, and subject P5-d, at 4.7 years of age, were found on echocardiography to have malformations of the aorta and ascending aorta. Subject P5-f was diagnosed with pulmonary valve stenosis at 40 years of age and subsequently underwent balloon valvuloplasty, with good postoperative recovery. These three individuals were classified as GD2. In contrast, the remaining affected family members exhibited no cardiac, pulmonary, or lens abnormalities and were therefore classified as having AD.

**Figure 2:**
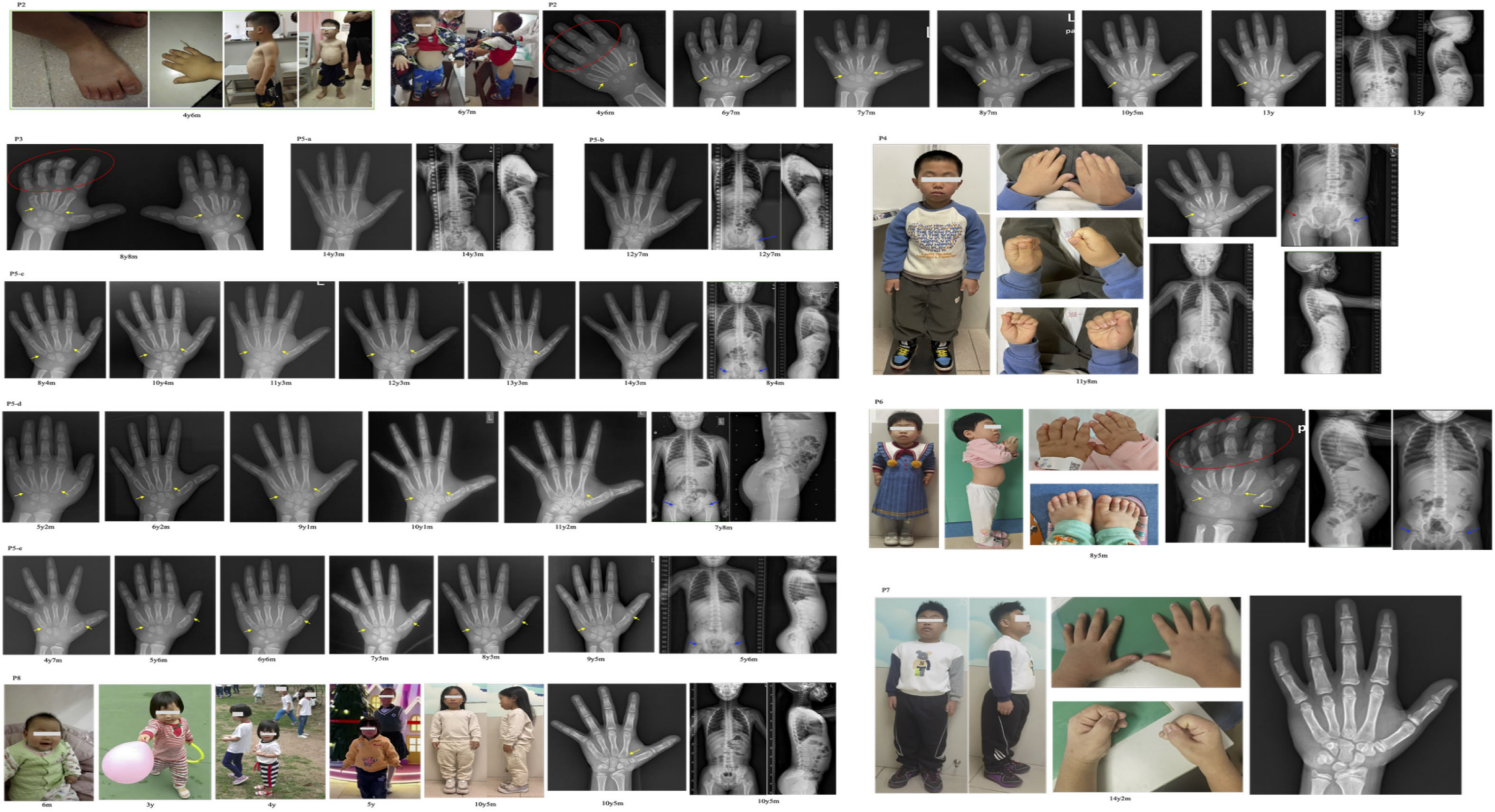
Facial features and radiographic findings of the hand and spine of the AD/GD2 participants. Red elliptical lines indicate conical epiphyses. Red arrows indicate femoral head epiphyseal dysplasia. Yellow arrows indicate the medial notch of the second metacarpal and the lateral notch of the fifth metacarpal. Blue arrows indicate beak-shaped femoral heads.

As shown in **Figure 2**, all the prepubertal and pubertal participants, except P1 (who did not undergo BA assessment), demonstrated delayed bone age accompanied by metacarpal shortening. Cone-shaped epiphyses were observed in participants P2, P3, and P6 (red ovals, **Figure 2**). Participant P4 demonstrated epiphyseal dysplasia of the right femoral head (red arrow, **Figure 2**). Medial notching of the second metacarpal and lateral notching of the fifth metacarpal (yellow arrows, **Figure 2**) were present in participants P2, P3, P4, P5-c, P5-d, P5-e, P6, and P8. Longitudinal imaging follow-up revealed that these notches diminished or resolved with age in participants P2, P5-c, P5-d, and P5-e. Beak-shaped femoral heads (blue arrows, **Figure 2**) were observed in participants P4, P5-b, P5-c, P5-d, P5-e, and P6. Notably, the typical ovoid vertebral bodies that are frequently reported in patients with AD or patients with GD2 were absent in this cohort.

Adenoid hypertrophy was observed in 21.4% (3/14) of the participants (P2, P3, and P4), all of whom underwent adenoidectomy. Participant P2 had recurrent purulent otitis media and underwent bilateral myringotomy with drainage at age 5 years. Participant P4 had moderate right-sided conductive hearing loss.

Among the 14 participants included in this cohort, 35.7% (5/14) exhibited skin thickening, whereas hepatomegaly was observed in two individuals (P1 and P3). Joint mobility limitation was present in 42.9% (6/14) of the participants (**Table 1**). Cardiopulmonary structural or functional abnormalities were identified in 50.0% (7/14) of the participants; all affected individuals were diagnosed with GD2 (**Table 1**). These abnormalities included pulmonary valve stenosis in two participants, severe mitral stenosis with pulmonary hypertension in one, isolated pulmonary hypertension in one, aortic root and ascending aortic abnormalities in two, and mixed ventilatory dysfunction with recurrent bronchopulmonary infections in one (**Table 1**).

### Genotype–phenotype correlation analysis

**Figure 3:**
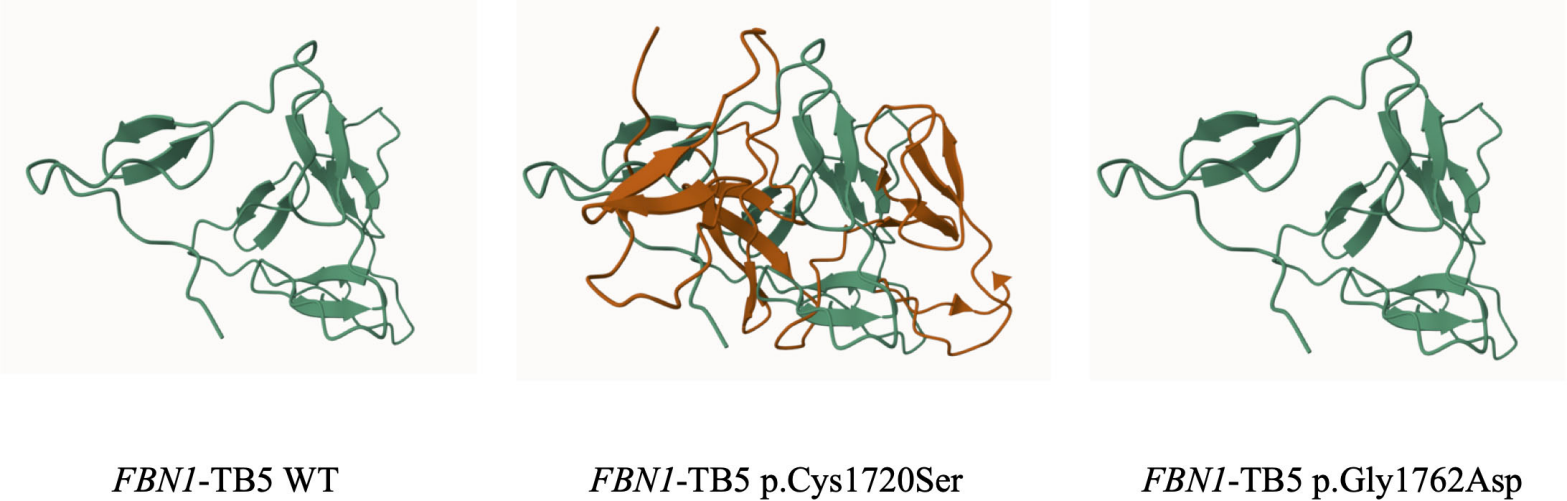
3D structural superposition of wild type and mutant FBN1 TB5 domain.

All participants in this cohort harbored heterozygous missense variants within the TB5 domain of the *FBN1* gene (**Table 1**). Two novel *de novo* variants of *FBN1* were identified: c.5159G>C (p.Cys1720Ser) in P6 and c.5285G>A (p.Gly1762Asp) in P7. For p.Cys1720Ser, the variant was not observed in large population databases, including gnomAD (v2.1.1, v3.1), 1000 Genomes Project, and East Asian reference datasets (PM2), occurred *de novo* in the proband with confirmed parentage (PS2), and was located within the TB5 domain, a mutational hotspot associated with AD/GD2(PM1). In silico prediction tools consistently suggested a deleterious effect, including REVEL (0.94), AlphaMissense (0.999), and PrimateAI (0.82) (PP3). Clinically, the patient presented with a phenotype highly consistent with FBN1-TB5–related acromelic dysplasia (PP4). Based on the combination of PS2, PM1, PM2, PP3, and PP4, this variant was classified as likely pathogenic according to ACMG/AMP guidelines. Consistent with this, our structural comparison demonstrates a marked conformational deviation from the wild-type model, supporting a substantial structural impact (**Figure 3**).

For p.Gly1762Asp, the variant was also absent from population databases (PM2) and occurred *de novo* (PS2). It is located within the TB5 domain (PM1), although in silico predictions were less consistent, with REVEL (0.59) indicating uncertain significance, while AlphaMissense (0.946) and PrimateAI (0.88) suggested a possible deleterious effect (PP3_supporting). The clinical phenotype was consistent with AD/GD2 spectrum disorder (PP4). However, given the weaker and partially conflicting computational evidence, this variant was classified as a variant of uncertain significance to likely pathogenic (VUS–LP range) under ACMG/AMP criteria, pending further functional validation. As shown in **Figure 3**, although no obvious global conformational disruption was observed in the predicted structure, this residue has been recurrently reported in pathogenic variants at the same position (e.g., p.Gly1762Ser and p.Gly1762Val) in patients with AD/GD2 spectrum disorders (8). In the 2011 study by Le Goff et al. (2), three patients with GD died at 9, 3, and 4 years of age, respectively; however, no detailed information regarding the causes of death was provided. In the 2021 study by Marzin et al. (7), a total of five patients with GD2 were reported to have died. Among them, four deaths were attributed to multilevel airway obstruction, including tracheomalacia, laryngomalacia, and bronchomalacia. Despite endoscopic interventions, the ages at death were 0.7, 3, 5, and 20 years, respectively. An additional patient died at 0.5 years of age, although the specific cause of death was not reported. One patient with AD died at 6 years of age in the context of pulmonary hypertension and asthma; however, the exact cause of death remained unclear.

In the deceased group, the median age at death was 4.0 years (IQR 1.9–7.5 years), whereas in surviving patients the median age was 14.0 years (IQR 9.0–22.0 years). As shown in **Table 2**, mortality was 4.0% (95% CI 0.7–19.6%) in the AD group and 29.6% (95% CI 15.9–48.5%) in the GD2 group (Wilson method, *P* = 0.038). To explore genotype–phenotype correlations, participants were stratified into three groups according to whether the *FBN1* TB5 variant altered the number of cysteine residues. Mortality differed significantly among the cysteine-reducing, cysteine-increasing, and unchanged groups. Pairwise comparisons revealed higher mortality in the cysteine-reducing group than in the unchanged group (*P* = 0.0015), whereas differences between the cysteine-reducing and cysteine-increasing groups (*P* = 0.2904) and between the cysteine-increasing and unchanged groups (*P* = 0.0942) were not significant. Further classification by amino acid substitution within TB5 revealed significant differences in mortality among cysteine substitutions, aromatic substitutions (e.g., tyrosine), and other substitutions (overall *P* < 0.001). Although mortality did not differ between the cysteine and aromatic groups (*P* = 0.5994), both groups had a greater mortality rate than the group with other substitutions (both *P* < 0.05).

**Table 2:** Mortality analysis between subgroups.

Group	No. of participants	Deaths, n (%, 95% CI)	P value^#^
Clinical subtype			
AD	25	1 (4.0%, 0.7-19.5%)	0.025
GD2	27	8 (29.6%, 15.9 -48.5%)	
Cysteine residue change (TB5 domain)			
Reducing	9	5 (55.6%, 26.7-81.1%)	
Increasing	19	4 (21.1%, 8.5-43.3%)	<0.001
Unchanged	24	0 (0.0%, 0.0-13.8%)	
Type of substituted amino acid (TB5 domain)			
Cysteine	9	5 (55.6%, 26.7-81.8%)	
Aromatic	16	4 (25.0%, 10.2-49.5%)	<0.001
Other amino acids	27	0 (0.0%, 0.0-12.5%)	

^#^Fisher’s exact test.

**Figure 4:**
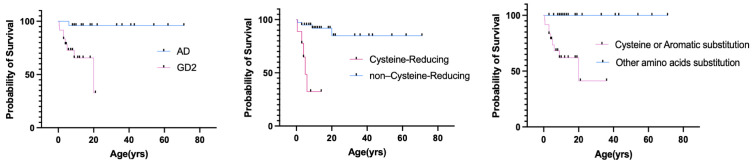
Kaplan-Meier curves of participants in different subgroups.

We extracted all available data from published cohorts (2, 7) and performed Kaplan–Meier survival analyses. The results (**Figure 4**) demonstrated that: (1) patients in GD2 subtype had significantly higher mortality compared with those in AD subtype (P = 0.00114); (2) *FBN1* variants associated with Cysteine-Reducing showed significantly increased mortality compared with non–Cysteine-Reducing variants (P < 0.0001); and (3) variants involving substitutions of cysteine or aromatic amino acids were associated with higher mortality than other amino acid substitution groups (P = 0.0002).

### Growth-promoting therapy

In this cohort, 11 participants received growth-promoting therapy. Participants P1 and P3 had previously undergone rhGH therapy for 2 years at external institutions (initiated at ages 6.0 and 4.5 years, respectively); both showed suboptimal responses, and detailed growth velocity (GV) data were unavailable. Participant P4 initiated rhGH therapy at age 5 years 7 months and achieved a GV of 8.1 cm/year over 1 year of treatment.

**Figure 5:**
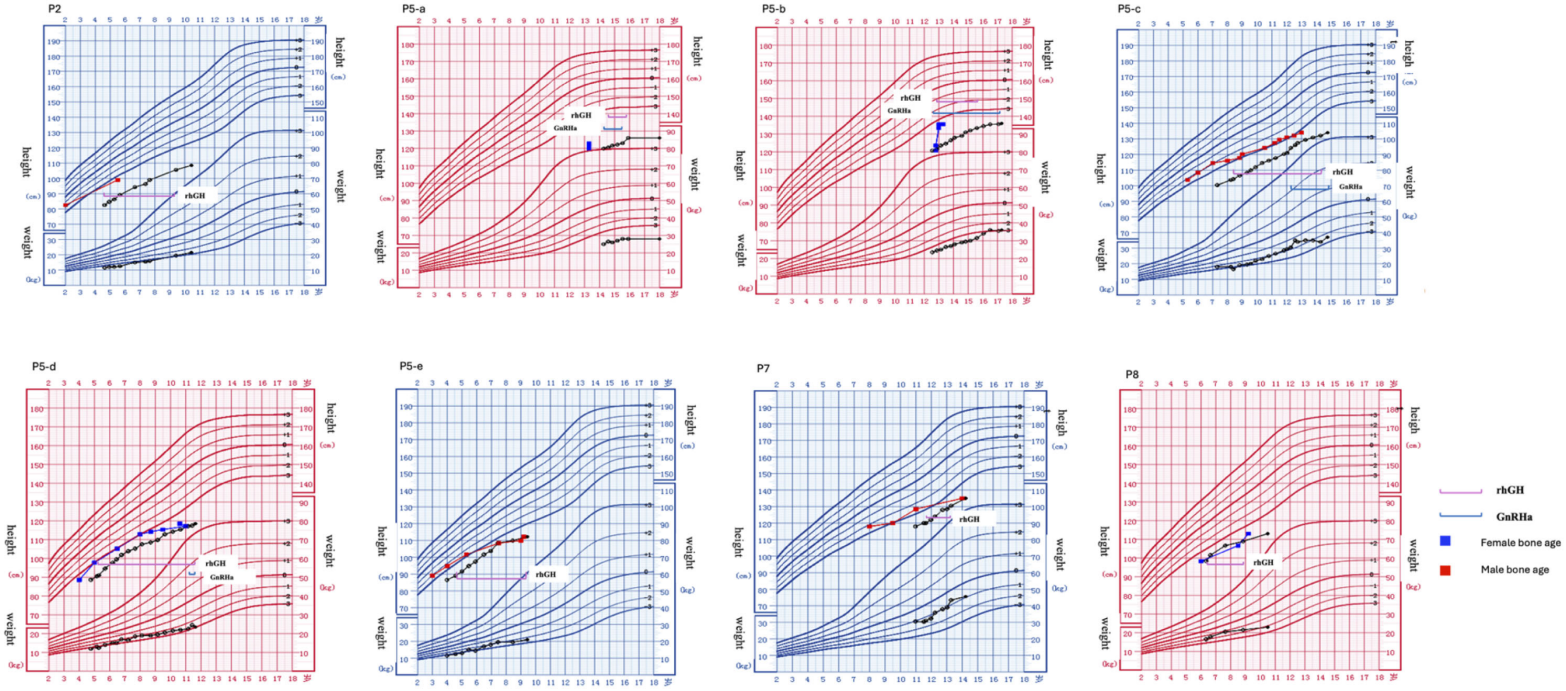
Growth curves of AD/GD2 participants receiving therapy at our center.

At our center, eight participants received growth-promoting therapy (**Table 3**, **Figure 5**). Following informed consent, treatment was initiated at a mean chronological age (CA) of 8.6 ± 4.0 years. Six prepubertal participants (P2, P5-c, P5-d, P5-e, P7, and P8) initiated rhGH therapy at a mean age of 6.9 ± 3.0 years. The starting rhGH dosage was 50 µg/kg/day, which was titrated to 66.7 µg/kg/day according to the clinical response. The mean growth velocity (GV) was 6.0 ± 1.1 cm/year in the first year, remained stable at 6.0 ± 1.0 cm/year in the second year, and declined to 3.5 ± 1.6 cm/year in the third year of treatment. Among the six prepubertal patients receiving rhGH, GV showed a similar pattern, at 6.0 ± 1.1, 6.0 ± 1.0, and 3.5 ± 1.7 cm/year across the first to third years, respectively.

**Table 3:** Clinical data of 8 AD/GD2 participants receiving growth-promoting therapy at our center.

Participant		P2	P5-a	P5-b	P5-c	P5-d	P5-e	P7	P8
Sex		M	F	F	M	F	M	M	F
Initiation of growth-promoting therapy	Age (years)	4.56	14.39	12.71	8.27	6.68	4.58	11.48	7.35
	Height (cm)/SDS	82.5/-6.34	119.9/-7.04	120.7/-5.7	104.3/-4.93	91.0/-6.13	89.0/-4.71	120.0/-4.06	101.5/-4.53
	Tanner stage	I	IV	III	I	I	I	I	I
	BA (years)	2.5	13.25	12.75	5.5	4	3	9.5	3.5
	IGF-1 (ng/mL)	42.6	312	204.6	116.1	43.1	52.4	178	127
Duration of GH therapy (years)		4.75	1.33	2.66	6	5.83	4.83	2	1.42
Duration of GnRHa therapy (years)		0	1.25	4.41	2	0.5	0	0	0
End of year 1	GV (cm/Y)	6.6	2.9	4.25	4.36	7.3	6	6.5	7
	Tanner stage/BA (years)	I/4	IV/13.3	III/13	I/6	I/5	I/4	II/10.5	I/4.5
	IGF-1 (ng/mL)	87.4	373	228.5	271.6	127.6	124	250	157.8
End of year 2	GV (cm/Y)	4.7		4.06	6.3	6.1	6.8	7	4.6
	Tanner stage/BA (years)	I/5.5		III/13	I/7	I/6	I/6	III/11.5	I/
	IGF-1 (ng/mL)	148		283.6	366	156.2	102	260	165.2
End of year 3	GV (cm/Y)	1.37		4.4	3.38	4.04	5.34		
	Tanner stage/BA (years)	I/6.5		III/13	II/8.5	I/7	I/7		
	IGF-1 (ng/mL)	147.5		330.0	115.0	118.0	157.0		
End of year 4	GV (cm/Y)	1.4		2.05	6.5	2.3	3.4		
	Tanner stage/BA (years)	I/7.5		III/13.25	III/10.5	I/7.5	I/9		
	IGF-1 (ng/mL)	100.2		358.0	255.0	108.2	160.0		
End of year 5	GV (cm/Y)	3.3			5.2	4.4			
	Tanner stage/BA (years)	I/			III/11.5	II/8.75			
	IGF-1 (ng/mL)				175	137			
End of year 6	GV (cm/Y)				2.7	2.2			
	Tanner stage/BA (years)				III/12.5	III/			
	IGF-1 (ng/mL)				136	110			

M, male; F, female; BA, bone age; GV, growth velocity.

Additional clinical details:P5-a: GnRHa 3 mo → +rhGH; stanozolol added after 1 yr for poor GV. P5-b: GnRHa 4 mo → +rhGH; stanozolol added after 3.5 yr. P5-c: rhGH 4 yr → +GnRHa. P5-d: rhGH 5.25 yr → +GnRHa. Compliance was poor for P5-a, P5-c, and P5-d.

Participants P5-a and P5-b were pubertal at diagnosis (BA 13.25 and 12.75 years, respectively) and initiated combined rhGH and GnRHa therapy at ages 14.39 and 12.71 years, respectively. Participant P5-a received GnRHa for 15 months, rhGH for 16 months, and stanozolol for 9 months and achieved a growth velocity (GV) of 2.9 cm/year and a final height of 127.5 cm (-6.13 SD). Participant P5-b received GnRHa for 53 months, rhGH for 32 months, and stanozolol for 18 months, with annual GVs of 4.25, 4.06, and 4.40 cm/year, respectively, and attained a final height of 139.5 cm (-3.91 SD).

Among the eight participants treated at our center, the mean duration of rhGH therapy was 3.6 ± 2.0 years, with a mean follow-up of 5.8 ± 1.7 years. No significant changes were observed in height-SDS, BMI-SDS, arm span-to-height ratio, or sitting height-to-height ratio before versus after treatment (all *P* > 0.05; **Table 4**). No abnormalities in glucose or lipid metabolism were detected during therapy. In the six participants who initiated rhGH before puberty, after 4.14 ± 1.95 years of treatment and 5.28 ± 1.67 years of follow-up, height-SDS remained stable at baseline and last follow-up [-5.02 (-4.49 ~-4.26) vs. -5.16 (-4.59 ~ -4.23), P = 0.60].

**Table 4:** Physical parameters before and after growth-promoting treatment in 8 AD/GD2 participants.

Parameter	Before rhGH treatment	Last follow-up	Z value*	P value
Age (years)	8.6 ± 4.0	13.89 ± 4.53		<0.001
Height-SDS	-4.6 (-5.3~-4.2)	-4.5 (-5.3~-4.3)	-1.400	0.161
BMI-SDS	0.59 ± 0.91	0.27 ± 0.80		0.306
Arm span/height	0.98 (0.94~0.99)	0.96 (0.94~0.96)	-1.363	0.173
Sitting height/height	0.59 ± 0.02	0.58 ± 0.01		0.336

*Wilcoxon signed-rank test. All of the other comparisons were performed by using paired t tests.

## Discussion

This study characterized the clinical and molecular features of 14 Chinese patients with FBN1-related acromelic dysplasia. In addition to having severe disproportionate short stature with marked acromelic involvement, the patients exhibited delayed bone age, metacarpal shortening, cone-shaped epiphyses, beak-shaped femoral heads, and variable multisystem involvement, including upper airway disease, otitis media, skin thickening, hepatomegaly, and cardiopulmonary abnormalities. Cardiopulmonary involvement was more frequently observed in patients with GD2 and may be associated with greater mortality compared with that in patients with AD. Analysis of TB5 domain variants suggested that substitutions involving cysteine or aromatic residues may be associated with an increased risk of mortality. Growth-promoting therapy may help maintain growth velocity during the early phase of treatment; however, long-term follow-up indicates that it does not significantly improve height-SDS.

Variants in the *FBN1* gene exhibit substantial molecular and phenotypic heterogeneity. Disruption of the extracellular microfibrillar network and dysregulated activation of transforming growth factor-β (TGF-β) signaling are central mechanisms underlying the spectrum of *FBN1*-related disorders, including both Marfan syndrome (MFS) and AD/GD2. While pathogenic variants associated with MFS are distributed throughout the gene (3), missense variants causing AD/GD2 are highly enriched within exons 41 and 42 (2), corresponding to the TB5 domain. Notably, TB5 variants can give rise to divergent phenotypes, with truncating variants more often linked to MFS (3) and missense variants predominantly associated with AD/GD2 (9). Emerging evidence indicates that even within TB5, specific missense variants may lead to contrasting clinical manifestations (8), underscoring the complexity of genotype–phenotype correlations. Collectively, these findings suggest that both the positional context and the biochemical properties of *FBN1* variants critically influence microfibrillar integrity and downstream TGF-β signaling, thereby shaping the disease phenotype.

Patients with AD and GD2 share core clinical features, including severe short stature, brachydactyly, joint contractures, and skin thickening, whereas patients with GD2 more frequently present with cardiopulmonary involvement. Despite these distinctions, the substantial overlap in molecular etiology and clinical presentation supports the concept that AD and GD2 represent a phenotypic continuum rather than distinct entities.

This notion is further supported by the intrafamilial variability observed in our cohort. Family P5 comprises seven affected individuals (P5-a to P5-g). Stratified by developmental stage, three pediatric patients (P5-c, P5-d, and P5-e) showed relatively similar height SDS values ranging from -4.0 to -5.0 SD. In contrast, two adolescent patients (P5-a and P5-b) exhibited greater variability in stature, with height SDS values of -5.32 and -6.81 SD, respectively. Among the two adult patients (P5-f and P5-g), the height SDS values were -5.52 and -4.00 SD, indicating a degree of phenotypic divergence comparable to that observed in adolescence. Within the P5 pedigree, all affected individuals harbored the same *FBN1* c.5099A>G variant but exhibited phenotypes ranging from those associated with AD to those associated with GD2. Similar observations have been reported; specifically, Tian et al. (2024) described a Chinese family in which members exhibited either AD or GD2 due to the same *FBN1* mutation (c.5179C>T) (10), and Le Goff et al. identified TB5 domain variants (c.5096A>G and c.5182G>A) that are responsible for both AD and GD2 across different populations (2).

Family P5 represents one of the most informative observations in our cohort, as individuals carrying the identical *FBN1* p.Tyr1700Cys variant exhibited a broad phenotypic spectrum ranging from AD to GD2. This observation is consistent with the emerging concept that *FBN1*-related disorders represent extracellular matrix (ECM) network diseases with multi-layered regulation rather than simple monogenic trait–phenotype relationships (11, 12). Emerging evidence supports the concept that fibrillinopathies are not strictly monogenic disorders with linear genotype–phenotype relationships, but rather complex ECM network diseases in which multiple biological layers contribute to phenotypic expression. Genetic modifiers affecting ECM assembly, microfibril stability, and TGF-β signaling have been increasingly recognized as important contributors to clinical variability (13, 14). In particular, variation in genes encoding latent TGF-β binding proteins (LTBPs), ADAMTS/ADAMTS-like proteins, fibulins, and other ECM regulators may influence microfibril architecture and downstream signaling output (11, 12). This is consistent with previous evidence in Marfan syndrome showing that genetic background can significantly modify disease severity, including aortic outcomes (13).

In addition to genetic modifiers, domain-specific properties of *FBN1* may also contribute to phenotypic heterogeneity (8, 15). The TB5 domain has been identified as a functional hotspot associated with a spectrum of disorders ranging from Marfan syndrome to GD2 and AD (8, 15). Structural and functional studies suggest that variants within key regulatory domains may affect long-range microfibril assembly and growth factor–related interactions (16). Importantly, identical amino acid substitutions may therefore result in variable phenotypes depending on microfibril assembly context, ECM composition, and tissue-specific molecular interactions (12).

Furthermore, fibrillin-1 is an integral component of a highly interconnected extracellular matrix network (matrisome) (12), in which perturbations of a single component may propagate through multiple structural and signaling pathways (11, 12). This network-based behavior provides a plausible biological framework for intrafamilial phenotypic variability, even in the presence of identical causal variants. Finally, epigenetic regulation and developmental context may further contribute to phenotypic divergence through tissue-specific modulation of ECM gene expression and growth factor signaling pathways (11, 12, 17).

Taken together, the phenotypic variability observed in Family P5 supports a multifactorial model in which *FBN1*-related acromelic dysplasia is shaped not only by the primary pathogenic variant, but also by genetic modifiers, ECM network interactions, and developmental or environmental influences. This supports the view that AD and GD2 represent a phenotypic continuum rather than strictly distinct entities.

Importantly, our findings highlight that AD/GD2 should not be regarded solely as a skeletal dysplasia characterized by short stature (18). In addition to having typical craniofacial features and brachydactyly, patients frequently exhibit multisystem involvement, including airway obstruction (e.g., adenoid hypertrophy), recurrent otitis media and conductive hearing loss, skin thickening, joint limitation, hepatomegaly, and cardiopulmonary abnormalities. At the molecular level, *FBN1* TB5 domain variants disrupt microfibrillar architecture and enhance TGF-β signaling, leading to impaired extracellular matrix integrity and dysregulated tissue remodeling (15, 19, 20). These mechanisms likely account for the broad systemic manifestations observed in our cohort. Thus, AD/GD2 is better conceptualized as a multisystem connective tissue disorder rather than an isolated skeletal condition.

Approximately half of the participants in our cohort were classified as overweight or obese, with a higher prevalence in GD2. This observation is consistent with prior reports. In Marzin et al.’s cohort of 38 AD or GD2 participants (7), GD2 participants had a mean height-SDS of -4.6 and mean weight-SDS of -2.5, whereas AD participants had a mean height-SDS of -5.2 and mean weight-SDS of -2.0. Notably, mean weight-SDS often exceeds mean height-SDS in these patients, resulting in an elevated BMI-SDS that may reflect a relatively robust or pseudomuscular body composition rather than true adiposity.

We identified two novel *FBN1* variants, c.5159G>C (p.Cys1720Ser) and c.5285G>A (p.Gly1762Asp), thereby expanding the mutational spectrum of TB5-associated AD/GD2. The TB5 domain contains eight highly conserved cysteine residues that are thought to form intradomain disulfide bonds, which are important for the proper folding and structural stability of fibrillin-1 (2, 21). The p.Cys1720Ser variant may affect one of these conserved cysteine residues (2) and might therefore potentially interfere with disulfide bond formation within the TB5 domain. In contrast, the pathogenic relevance of p.Gly1762Asp remains less clear; however, this residue is located within the highly conserved TB5 domain of fibrillin-1, which is evolutionarily conserved and contains residues that are likely important for maintaining domain structure and stability (21, 22).

Our genotype–phenotype analysis revealed that variants affecting key structural residues within the TB5 domain may be associated with worse clinical outcomes. Substitutions leading to loss of cysteine residues were associated with the higher mortality rates, likely reflecting disruption of disulfide bond formation, a key determinant of fibrillin-1 structural integrity, as previously demonstrated in fibrillinopathies (2, 21). Similarly, variants involving aromatic residues were associated with increased mortality. Aromatic amino acids play a critical role in stabilizing the hydrophobic core and β-sheet architecture of the TB5 domain; their substitution may alter the local chemical environment and impair domain folding (2, 21). Cryo-EM structural analysis by Godwin et al. (2023) further demonstrated that TB5 mutations not only disrupt local heparan sulfate (HS) binding but also induce long-range structural effects that alter the overall conformation of microfibrils (16). As the TB5 domain represents a known mutational hotspot, it may be particularly sensitive to such structural perturbations, thereby contributing to impaired microfibril assembly. However, these proposed mechanisms remain hypothetical and require further structural and functional validation.

Patients carrying cysteine or aromatic residues substitutions may represent a higher-risk subgroup. At the time of diagnosis, more detailed prognostic counseling should be provided to caregivers. During follow-up, closer surveillance is recommended, including more frequent clinical assessments, particularly of cardiopulmonary function. Although these observations are preliminary and are primarily based on genotype–phenotype correlation analyses derived from published retrospective cohorts, they may nonetheless support risk stratification and individualized management of FBN1 TB5-related disorders.

Growth hormone therapy in our cohort had a partial but clinically relevant effect. While no significant improvement in height-SDS was observed in a long term follow-up, growth-promoting treatment appeared to maintain growth trajectories parallel to standard growth curves during the first two years of therapy. The he growth velocity decreased thereafter, likely reflecting both suboptimal adherence in some cases and intrinsic limitations imposed by the underlying disease biology. These findings suggest that growth-promoting therapy in *FBN1*-related AD/GD2 may confers partial efficacy, but fails to induce the catch-up growth typically observed in conditions such as growth hormone deficiency or small for gestational age. Mechanistically, persistent activation of TGF-β signaling and microfibrillar disorganization may impair chondrocyte maturation and endochondral ossification, thereby limiting the full anabolic effects of GH on the growth plate (2, 23, 24).Interindividual variability in response to rhGH was evident. Participants treated at younger chronological ages and bone ages, as well as those with longer treatment durations, appeared to derive greater benefit, suggesting that early intervention may be advantageous. Consistent with prior reports, height-SDS improvements in the literature range from minimal change to modest gains (0.4–1.6 SD) (9, 25–27), highlighting heterogeneity in treatment response. Differences in genotype, disease severity, treatment timing, dosing strategies, and concomitant therapies likely contribute to these variable outcomes.

Long-term follow-up further suggested that certain skeletal abnormalities may have partially improved over time in our cohort, indicating a degree of structural plasticity. However, given the progressive and multisystem nature of the disease, continued longitudinal monitoring remains essential.

## Strengths and limitations

This study provides a detailed phenotypic characterization and relatively long-term follow-up of growth-promoting therapy in a rare cohort of patients with *FBN1*-related AD/GD2.

However, several limitations should be acknowledged. The sample size was small and derived from a single center, introducing potential selection bias. In addition, treatment was not standardized, with variability in rhGH dosing and the timing of initiation, duration, and use of adjunctive therapies, which may confound the interpretation of efficacy. Larger, prospective, multicenter studies are needed to better define optimal management strategies.

A further limitation is inter-cohort heterogeneity arising from the integration of previously published datasets. Differences in clinical assessment, variant annotation, and outcome definitions may introduce variability and bias in genotype–phenotype and survival analyses. Moreover, the retrospective design and reliance on published data limited the completeness and consistency of key variables, including phenotypic characterization, follow-up information, and cause-of-death reporting. Therefore, these findings should be interpreted as exploratory and hypothesis-generating. Prospective multicenter studies with standardized phenotyping, harmonized variant annotation, and uniform longitudinal follow-up are required to validate these observations.

## Summary

This study of 14 patients with *FBN1*-related AD/GD2 demonstrated that TB5-domain missense variants underlie a multisystem disorder characterized by severe short stature, brachydactyly, and frequent cardiopulmonary involvement. Variants affecting cysteine or aromatic residues may associated with increased mortality, highlighting the importance of the structural integrity of fibrillin-1. Growth hormone therapy showed partial efficacy by maintaining growth velocity but did not result in catch-up growth. These findings emphasize the role of genotype in risk stratification and management. Growth promoting therapy appeared to show partial efficacy in maintaining growth velocity but did not clearly result in catch-up growth. These findings raise the possibility that genotype may be involved in risk stratification and clinical management, although further validation is needed.

## Data Availability

The raw data supporting the conclusions of this article will be made available by the authors, without undue reservation.
